# News from Societies

**Published:** 1959-07

**Authors:** 


### *News from Societies*

### PROGRAMMES FOR 1959–60 SESSION

### BRISTOL MEDICO-CHIRURGICAL SOCIETY

*Secretary:* Dr. B. E. McConnell, 4 Napier Road, Bristol 6

14th Oct.: **Annual General Meeting. Presidential Address.** F. W. Terrell Hughes, Esq., M.B., CH.B.

11th Nov.: **Clinical Meeting.** Frenchay Hospital.

9th Dec.: **Address** by Alan Moncrieff, Esq., C.B.E., M.D., F.R.C.P.

Further meetings will be announced in the next issue.

### THE DEVON AND EXETER MEDICO-CHIRURGICAL SOCIETY

*Secretary:* Dr. F. S. W. Brimblecombe, 6 Barnfield Crescent, Exeter.

15th Oct., at 3 p.m.: **Clinical Meeting.**

29th Oct., at 3.30 p.m.: Professor Max Rosenheim, C.B.E., M.D., F.R.C.P. **"The Treatment of Nephritis".** (A combined meeting with the B.M.A. (Exeter and South Western)). Dinner.

19th Nov., at 8.15 p.m.: Jack Simpson, Esq., M.D., M.R.C.P., **"Confusion and Dementia in the Elderly".**

10th Dec., at 8.15 p.m.: **Clinico-pathological Demonstration.**

7th Jan., at 8.15 p.m.: Dr. Sheila Sherlock, M.D., F.R.C.P., **"Advances in the Treatment of Liver Disease".**

28th Jan., at 8.15 p.m.: **Clinical Meeting** at Princess Elizabeth Orthopaedic Hospital.

18th Feb., at 8.15 p.m.: J. Romilly Withycombe, Esq., M.S., F.R.C.S., **"Changing Trends in Urological Surgery".**

17th Mar., at 3 p.m.: **Clinical Meeting.** (Council Meeting to follow).

21st Apr., at 8.15 p.m.: **Annual General Meeting** and **Clinico-pathological Demonstration.**

### B.M.A. (Bristol Division)

*Secretaries:* Dr. A. S. Anderson, 10 Leigh Road, Bristol 8, and
Dr. H. Temple Phillips, Berkeley House, Charlton Road, Westbury-on-Trym, Bristol.

9th Oct., **Annual Dance,** Grand Spa Hotel, Clifton.

18th Oct., at 2.45 p.m.: **St. Luke's Tide Service** in Bristol Cathedral. Preacher: The Right Reverend the Bishop of Bristol.

18th Nov., at 8.30 p.m.: Ian D. Grant, Esq., M.D., **"Medical Services and Conditions in Australia and New Zealand".** At the Royal Fort.

3rd May, at 8 p.m.: **Informal Reception** at Ashton Court Country Club.

4th May, at 8.30 p.m.: **Annual General Meeting** at the Royal Fort.

25th May: (Provisional date). **Clinical Meeting** at B.R.I.

10th June (Provisional date). **Reception for newly qualified doctors.** In the Garden Hall, Royal Fort.

Other meetings to be arranged.

### B.M.A. (Cornwall Division)

*Secretary:* Dr. E. Townsend, Fieldways, Tregenna Lane, Camborne.

26th Nov.: **Annual Dinner and Dance,** Hotel Bristol, Newquay.

April: **B.M.A. Clinical Lecture.** Royal Cornwall Infirmary, Truro. (Details still under consideration.)

### B.M.A. (Gloucestershire Branch)

*Secretary:* Dr. H. G. Scott-Kerr, 29 Leckhampton Road, Cheltenham.

8th Oct.: **Presidential Address.** C. de Vere Shortt, Esq., B.A., M.D., B.CH., B.A.O., **"Operation Overlord".** Cheltenham.

12th Nov.: **Clinical Meeting.** Representatives' Report, Annual Report and Financial Statement. Gloucester.

10th Dec.: **Address.** Sir Thomas Lund, C.B.E., Secretary of the Law Society, **"The Law of Negligence as it Affects the Medical Profession".** A Joint Meeting with the Gloucestershire Members of the Gloucestershire and Wiltshire Incorporated Law Society. Cheltenham.

14th Jan.: Paper. J. Lomas, Esq., B.A., M.B., B.CHIR., D.P.M., **"Tranquillizers in Psychiatry".** Gloucester.

11th Feb.: **"A Brains Trust".** Members of the Team: Four Gloucestershire Consultants. Cheltenham.

10th Mar.: Paper and Film. P. Boreham, Esq., M.CHIR., F.R.C.S., **"Indications for Prostatectomy".** Gloucester.

14th Apr.: **B.M.A. Lecture.** The Right Hon. W. S. Morrison, P.C., M.C., Q.C., M.P., Speaker of the House of Commons, **"The Doctor in Literature".** (Doctors' Ladies to be invited). Cheltenham.

12 May.: **Annual Meeting and Paper.** J. D. Riley, Esq., M.B., CH.B., **"The Medical Aspects of Motoring".** Winchcombe.

9th June: **President's Guest.** Brig. H. L. Glyn Hughes, C.B.E., D.S.O., M.C., O.H.P. Cheltenham.

Unless otherwise stated, the meetings will be held at 6.15 p.m. The place of the meetings will be stated in the circular notices.

### B.M.A. (South-Western Branch)

*Secretary:* Dr. F. E. Graham-Bonnalie, St. John's House, 37 The Strand, Topsham, near Exeter.

25th Oct.: Exeter Division Golf Meeting, Dawlish Warren.

29th Oct., at 3.30 p.m.: **Combined Meeting** with the Exeter and South Devon Medico-Chirurgical Society. Speaker: Professor Max Rosenheim, C.B.E., M.D., F.R.C.P. Library, Royal Devon and Exeter Hospital. Followed by **Annual Dinner.**

7th Nov.: South-Western Branch Autumn **Clinical Meeting** at the Infirmary, Barnstaple.

### BRISTOL SOUTH MEDICAL GROUP

*Secretary:* Dr. H. I. Howard, 102 Church Road, Bishopsworth, Bristol 3.

28th Oct.: John Naish, Esq., M.A., M.D., F.R.C.P., **"Facts and Fancies in Medicine".**

25th Nov.: Discussion Group 1; Royal Commission, 2; Members' Cases.

16th Dec.: **"Wines"** by Harvey.

10th Jan.: Films of Medical Interest.

7th Feb.: Competition—Social Evening.

8th Mar.: **Dance** to be held at Ashton Court Country Club.

April: **Annual General Meeting and Supper.** (Date to be announced later).

### BRISTOL YOUNG DOCTORS' GROUP

*Secretary:* Dr. P. B. Bailey, Melba, Bristol Road, Whitchurch.

Meetings are held quarterly in the B.R.I. to discuss matters of topical interest.

### COSSHAM MEDICAL SOCIETY

*Secretary:* Dr. K. W. Moulding, 235 Lodge Causeway, Bristol

6th Oct.: **Presidential Address.** J. H. Duerden, Esq., M.B., CH.B.

3rd Nov.: Mrs. D. Vinter, **"Old Kingswood".**

1st Dec.: R. H. Allen, Esq., M.A., B.SC., Visit to Kingswood Training and Classifying School.

5th Jan.: McDonald Critchley, Esq., M.D., F.R.C.P., **"Language of Gesture".**

26th Jan.: **Annual Supper Dance,** Ashton Court Country Club.

2nd Feb.: Professor A. I. Darling, D.D.SC., F.D.S., R.C.S., **"Common Disorders of the Teeth and Mouth".**

1st Mar.: W. T. Cussen, Esq., M.B., B.CH., **"Athletic Injuries."**

5th Apr.: **Annual General Meeting.**

15th June, at 2.15 p.m.: Visit to Fry's Factory at Somerdale.

### BATH CLINICAL SOCIETY

*Secretary:* Dr. J. R. Bolton, 61 Englishcombe Lane, Bath.

10th Oct.: L. C. Hill, Esq., M.D., F.R.C.P., **"Pain".**

14th Nov.: **Clinical Meeting** at St. Martin's Hospital, Bath.

12th Dec.: J. Apley, Esq., M.D., F.R.C.P., **"The Evolution of Paediatrics".**

8th Jan.: H. Gibson, Esq., M.D., D. W. Pugh, Esq., M.D., F.R.C.P., T. L. Schofield, Esq., F.R.C.S., **Symposium on the Use of Anticoagulants.**

12th Feb.: **Joint Meeting** with the Bath Law Society at St. Martin's Hospital, Bath, to discuss the new Mental Health Act.

11th Mar.: L. H. Savin, Esq., M.D., M.S., M.R.C.P., F.R.C.S., Senior Ophthalmic Surgeon King's College Hospital, **"Facial Appearances in Disease".**

8th Apr.: **Clinical Meeting.**

23rd Apr.: **Annual Dinner** at Fortt's Restaurant.

All meetings will be held at the Royal United Hospital, Bath, unless otherwise stated.

### CORNWALL CLINICAL SOCIETY

*Secretaries:* Dr. J. D. Hardy, Royal Cornwall Infirmary, Truro; and Dr. B. A. Gwynne Jenkins, Central Chest Clinic, Tuckingmill, Cornwall.

1st Oct.: J. H. Sheldon, Esq., C.B.E., M.D., F.R.C.P., **"Disturbance of Postural Control in Old Age".** Royal Cornwall Infirmary.

6th Nov.: **Clinical Evening.** Camborne-Redruth Hospital.

26th Nov.: **B.M.A. Dinner and Dance.** Newquay.

3rd Dec.: Professor R. A. Willis, M.D., F.R.C.P., **"Tumours in Infancy and Childhood".** Royal Cornwall Infirmary.

8th Jan.: **Clinical Evening or Quiz.** Camborne-Redruth Hospital.

4th Feb.: C. T. Andrews, Esq., M.D., F.R.C.P., **"The Story of Florence Nightingale".** Royal Cornwall Infirmary. (Ladies' Night).

4th Mar.: **Clinical Evening.** St. Lawrence's Hospital, Bodmin.

7th Apr.: **B.M.A. Clinical Lecture.** Royal Cornwall Infirmary.

6th May: **Clinical Evening.** Camborne-Redruth Hospital.

2nd June: **Presidential Address and Annual General Meeting.** Royal Cornwall Infirmary.

### ROYAL DEVON AND EXETER HOSPITAL POSTGRADUATE PROGRAMME

*Secretary:* Mr. H. Dendy Moore, 4 Magdalen Road, Exeter.

The Exeter Postgraduate Meetings will take place on Tuesdays at 8.30 p.m. from 13th October to 8th December.

### SOCIETY OF MEDICAL OFFICERS OF HEALTH WEST OF ENGLAND BRANCH

*Secretary:* Dr. R. H. G. H. Denham, Council Offices, 8 Cleveland Place East, Bath.

The Society is divided into two sections, Northern and Southern. Each section holds four meetings a year, one of which being a joint meeting of both sections.

3rd Oct.: **Presidential Address.** G. F. Bramley, Esq., M.D., D.P.H., M.O.H., Gloucestershire. Taunton.

2nd Jan.: At Bristol. (Details not yet available).

2nd Apr.: At the Pump Room, Bath. (Details not yet available).

May: **Joint Meeting** of Northern and Southern Sections. (Details not yet available).

### SOUTH SOMERSET MEDICAL CLUB

*Secretary:* Dr. D. M. Barry, X-Ray Dept., Yeovil General Hospital.

15th Oct.: J. F. Davidson, Esq., O.B.E., M.B., D.P.H., **"Public Health".**

19th Nov.: George D. Kersley, Esq., M.D., F.R.C.P., **"Rheumatic Diseases".**

21st Jan.: **Dinner and Dance.**

18th Feb.: J. A. Boycott, Esq., M.A., D.M., **"Infectious Disease in Boarding Schools".**

17th Mar.: Symposium. **"Local Medical Services".**

18th Apr.: K. C. Bailey, Esq., M.D., D.P.M., **"Mental Health Acts".**

### TORQUAY AND DISTRICT MEDICAL SOCIETY

*Secretaries:* Dr. D. K. MacTaggart, Health Department, St. Marychurch Town Hall, Torquay; and Dr. J. B. Taylor, Corner Place, Dartmouth Road, Paignton.

29th Oct., at 4 p.m.: Sir Ralph Marnham, K.C.V.O., M.CHIR., F.R.C.S., **"Antibiotics, their Use and Abuse"**, at 7.30 p.m.: **Dinner and Dance** at the Palace Hotel, Torquay.

12th Nov.: S. Cochrane Shanks, Esq., M.D., F.R.C.P., F.F.R., **"Medicine, Radiology and the Law".**

3rd Dec.: J. F. Dow, Esq., B.A., M.B., F.R.C.P., **"More Harm than Good".**

28th Jan.: Sir Arthur Porritt, K.C.M.G., K.C.V.O., O.B.E., CH.M., F.R.C.S., **"The Problem of Breast Cancer, Past, Present and Future".**

25th Feb.: R. Bodley Scott, Esq., M.A., D.M., F.R.C.P., **"Recent Advances in Blood Diseases".**

10th Mar.: Wylie McKissock, Esq., O.B.E., M.S., F.R.C.S., **"Acute Cerebro-Vascular Accidents".**

31st Mar.: R. H. Gosling, Esq., M.D., D.P.M., **"The General Practitioner and Psychiatry".**

19th May: **Annual General Meeting.** Victoria Hotel, Torquay.

Meetings are held at the Torbay Hospital at 8.30 p.m. unless otherwise stated.

### BRISTOL MEDICAL DRAMATIC CLUB

The next production will be **"The Ghost Train"** by Arnold Ridley at the Y.M.C.A. Hall in Trenchard Street, during the week beginning 30th November, 1959. Details and tickets may be obtained from Dr. P. B. Bailey, Whitchurch. Production by Dr. S. Curwen.

